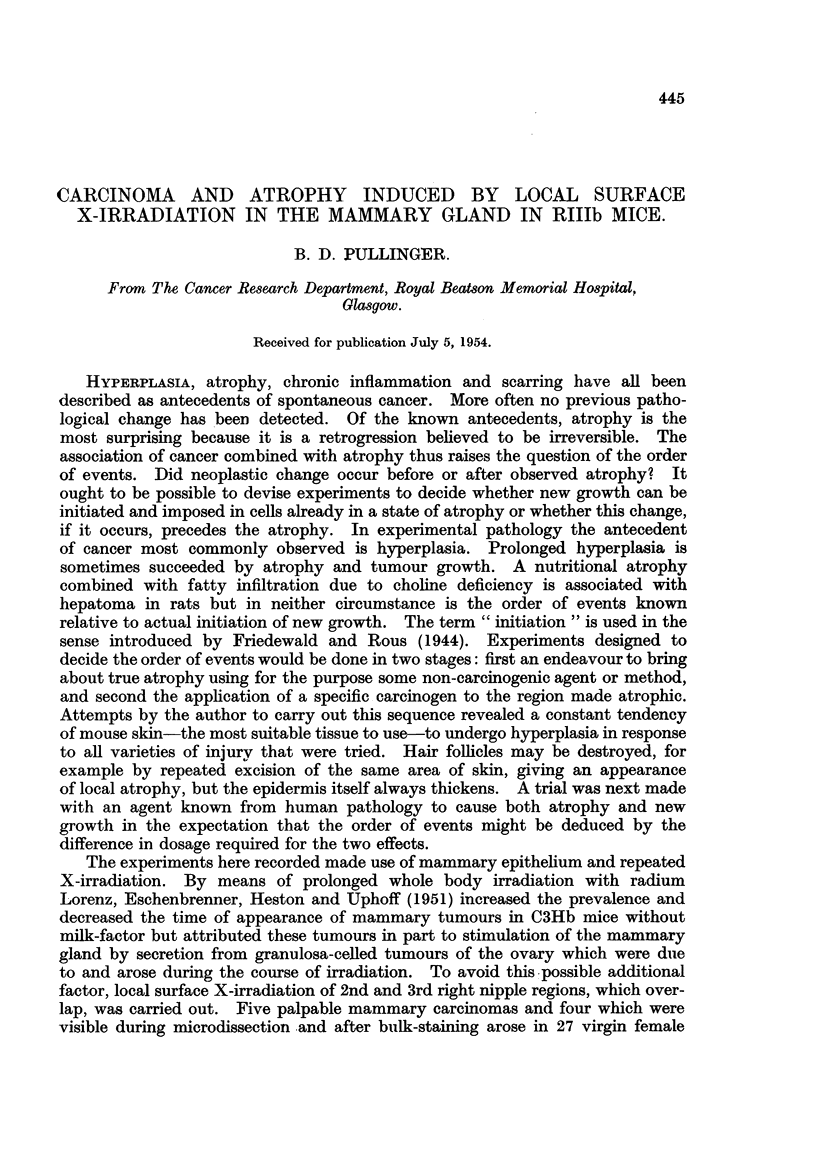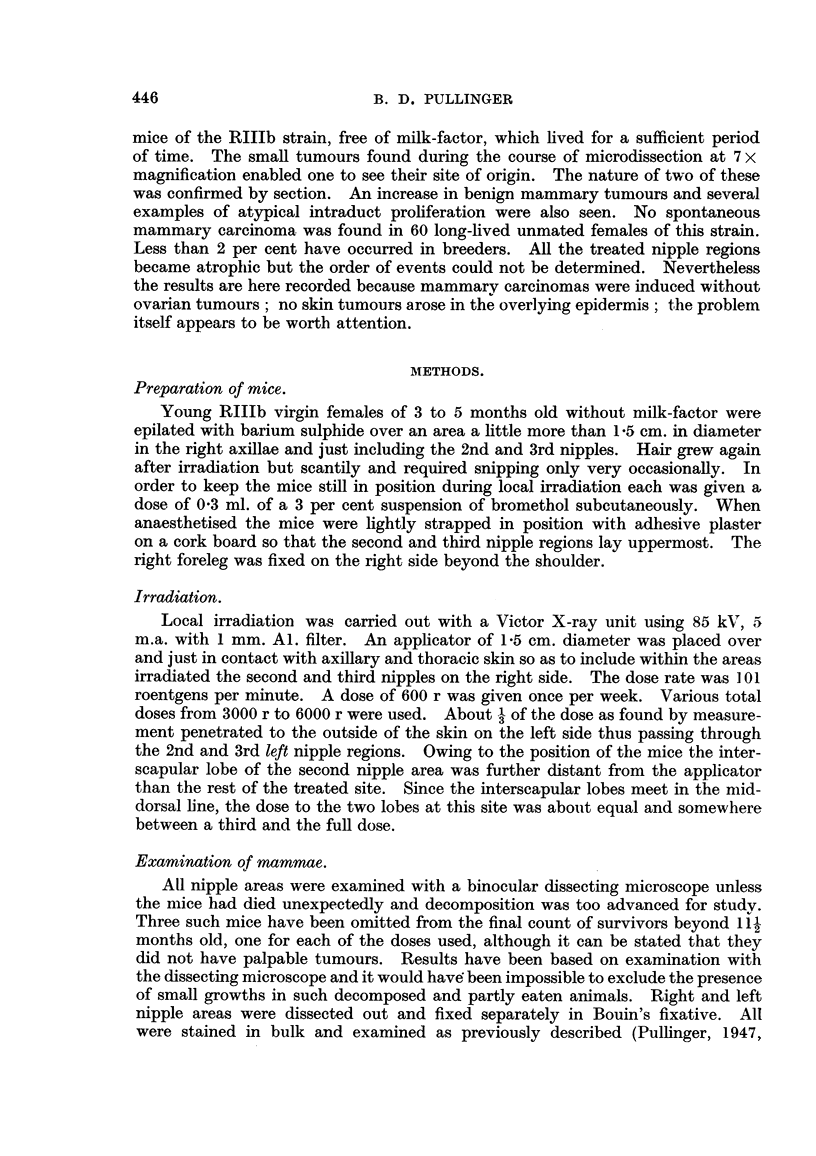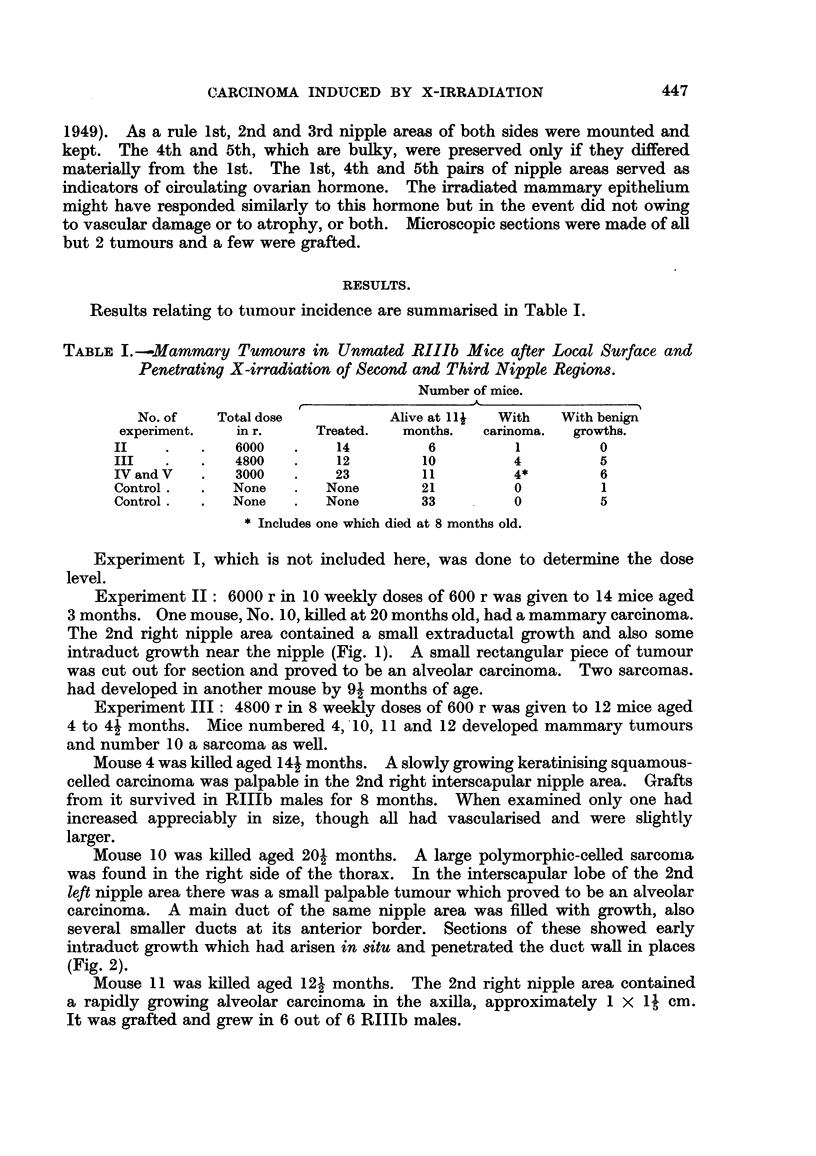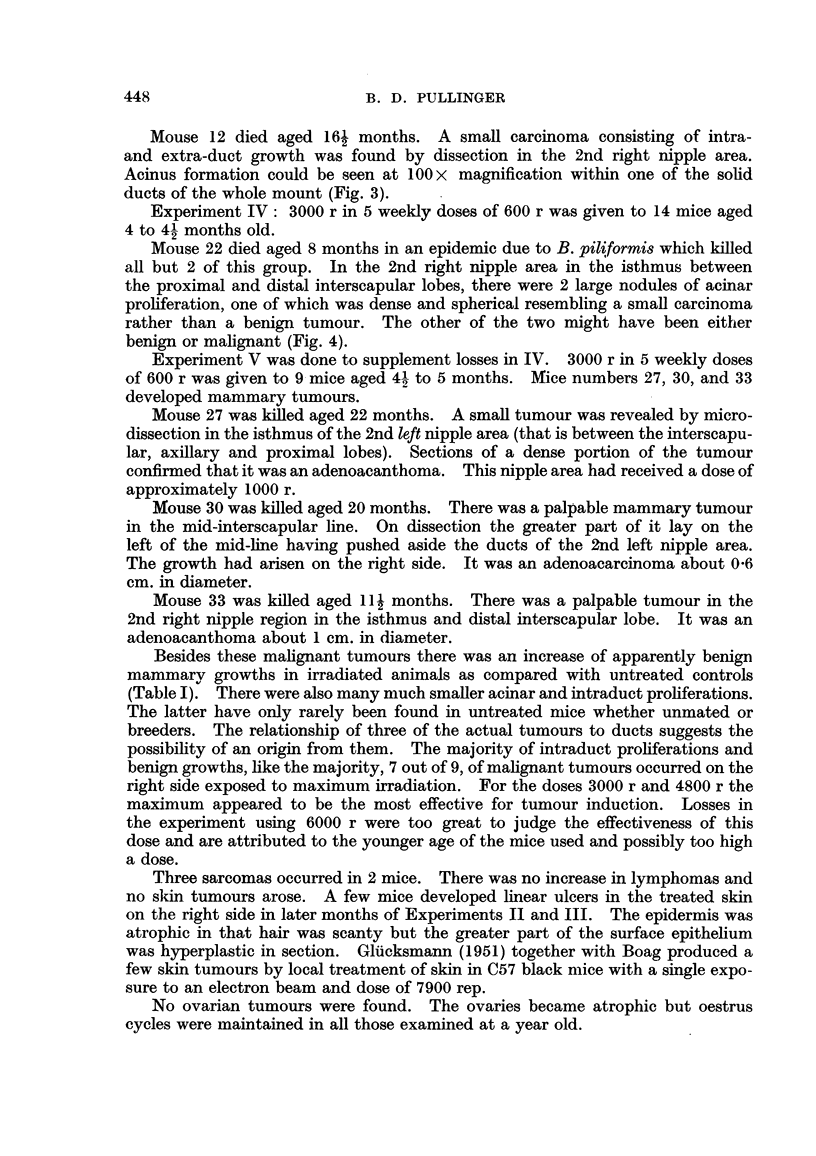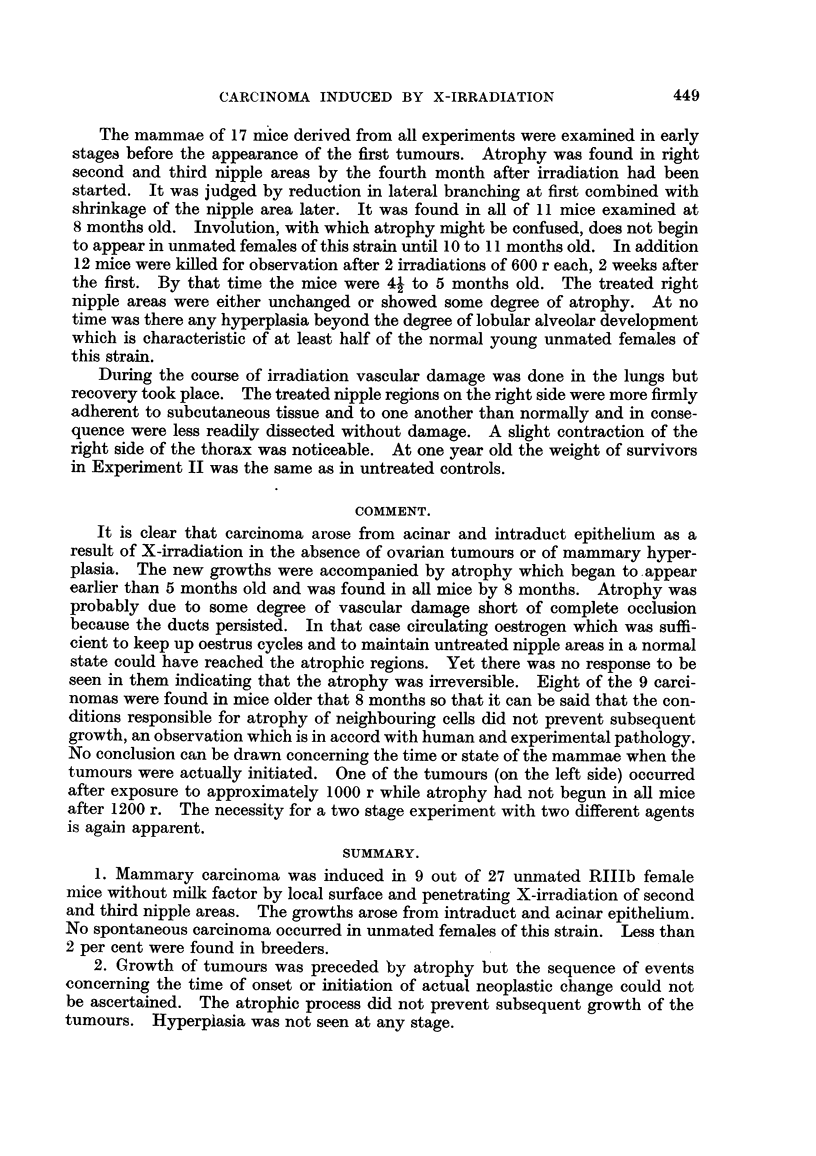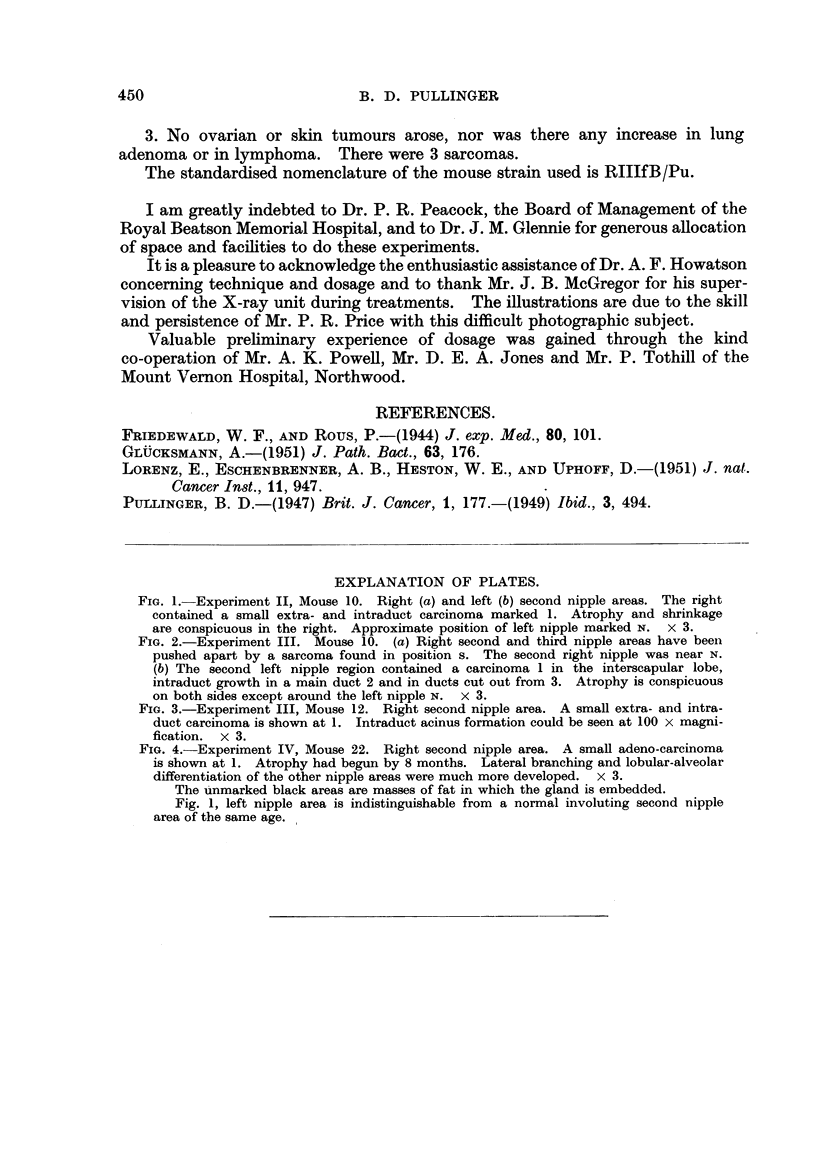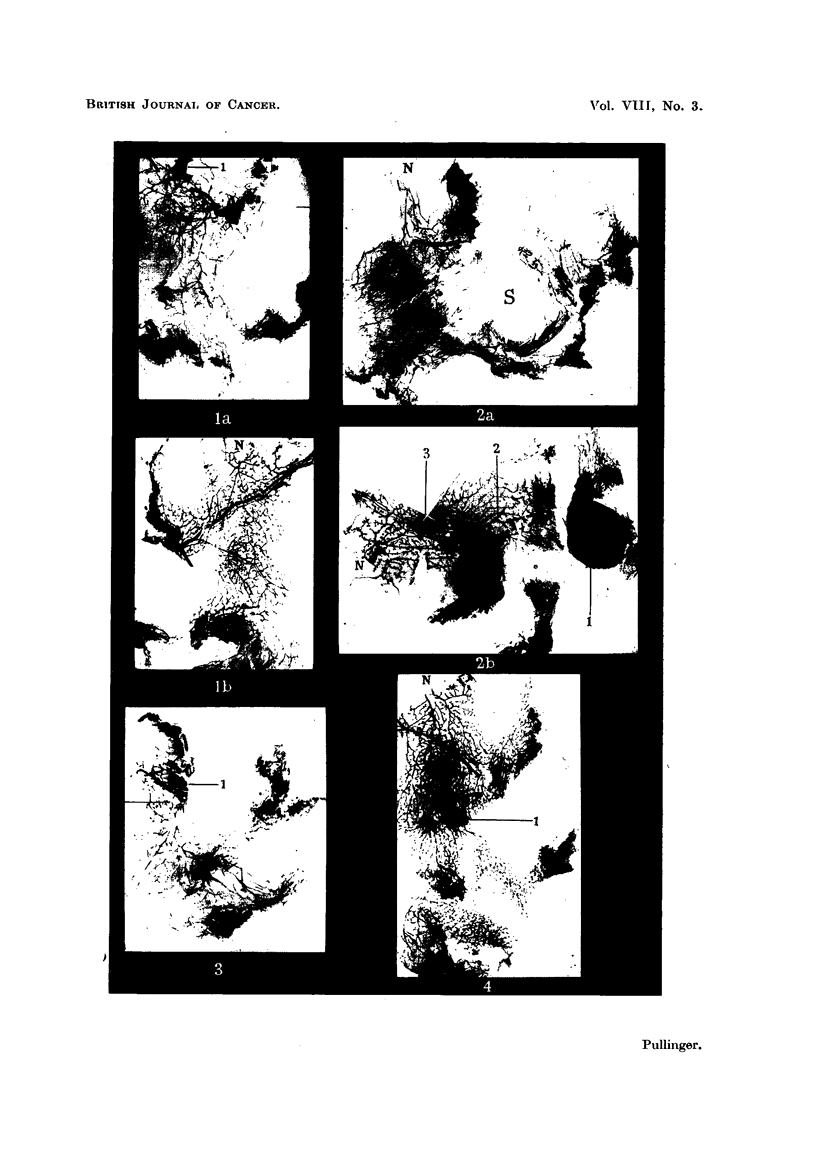# Carcinoma and Atrophy induced by Local Surface X-Irradiation in the Mammary Gland in RIIIb Mice

**DOI:** 10.1038/bjc.1954.47

**Published:** 1954-09

**Authors:** B. D. Pullinger

## Abstract

**Images:**


### CARCINOMA AND ATROPHY INDUCED BY LOCAL SURFACE X-IRRADIATION IN THE MAMMARY GLAND IN RIIIb MICE.

B. D. PULLINGER.

*From The Cancer Research Department, Royal Beatson Memorial Hospital, Glasgow.*

Received for publication July 5, 1954.

HYPERPLASIA, atrophy, chronic inflammation and scarring have all been described as antecedents of spontaneous cancer. More often no previous pathological change has been detected. Of the known antecedents, atrophy is the most surprising because it is a retrogression believed to be irreversible. The association of cancer combined with atrophy thus raises the question of the order of events. Did neoplastic change occur before or after observed atrophy? It ought to be possible to devise experiments to decide whether new growth can be initiated and imposed in cells already in a state of atrophy or whether this change, if it occurs, precedes the atrophy. In experimental pathology the antecedent of cancer most commonly observed is hyperplasia. Prolonged hyperplasia is sometimes succeeded by atrophy and tumour growth. A nutritional atrophy combined with fatty infiltration due to choline deficiency is associated with hepatoma in rats but in neither circumstance is the order of events known relative to actual initiation of new growth. The term " initiation " is used in the sense introduced by Friedewald and Rous (1944). Experiments designed to decide the order of events would be done in two stages: first an endeavour to bring about true atrophy using for the purpose some non-carcinogenic agent or method, and second the application of a specific carcinogen to the region made atrophic. Attempts by the author to carry out this sequence revealed a constant tendency of mouse skin—the most suitable tissue to use—to undergo hyperplasia in response to all varieties of injury that were tried. Hair follicles may be destroyed, for example by repeated excision of the same area of skin, giving an appearance of local atrophy, but the epidermis itself always thickens. A trial was next made with an agent known from human pathology to cause both atrophy and new growth in the expectation that the order of events might be deduced by the difference in dosage required for the two effects.

The experiments here recorded made use of mammary epithelium and repeated X-irradiation. By means of prolonged whole body irradiation with radium Lorenz, Eschenbrenner, Heston and Uphoff (1951) increased the prevalence and decreased the time of appearance of mammary tumours in C3Hb mice without milk-factor but attributed these tumours in part to stimulation of the mammary gland by secretion from granulosa-celled tumours of the ovary which were due to and arose during the course of irradiation. To avoid this possible additional factor, local surface X-irradiation of 2nd and 3rd right nipple regions, which overlap, was carried out. Five palpable mammary carcinomas and four which were visible during microdissection and after bulk-staining arose in 27 virgin female mice of the RIIIb strain, free of milk-factor, which lived for a sufficient period of time. The small tumours found during the course of microdissection at 7× magnification enabled one to see their site of origin. The nature of two of these was confirmed by section. An increase in benign mammary tumours and several examples of atypical intraduct proliferation were also seen. No spontaneous mammary carcinoma was found in 60 long-lived unmated females of this strain. Less than 2 per cent have occurred in breeders. All the treated nipple regions became atrophic but the order of events could not be determined. Nevertheless the results are here recorded because mammary carcinomas were induced without ovarian tumours ; no skin tumours arose in the overlying epidermis ; the problem itself appears to be worth attention.

### METHODS.

### *Preparation of mice.*

Young RIIIb virgin females of 3 to 5 months old without milk-factor were epilated with barium sulphide over an area a little more than 1·5 cm. in diameter in the right axillae and just including the 2nd and 3rd nipples. Hair grew again after irradiation but scantily and required snipping only very occasionally. In order to keep the mice still in position during local irradiation each was given a dose of 0·3 ml. of a 3 per cent suspension of bromethol subcutaneously. When anaesthetised the mice were lightly strapped in position with adhesive plaster on a cork board so that the second and third nipple regions lay uppermost. The right foreleg was fixed on the right side beyond the shoulder.

### *Irradiation.*

Local irradiation was carried out with a Victor X-ray unit using 85 kV, 5 m.a. with 1 mm. Al. filter. An applicator of 1·5 cm. diameter was placed over and just in contact with axillary and thoracic skin so as to include within the areas irradiated the second and third nipples on the right side. The dose rate was 101 roentgens per minute. A dose of 600 r was given once per week. Various total doses from 3000 r to 6000 r were used. About $\frac{1}{3}$ of the dose as found by measurement penetrated to the outside of the skin on the left side thus passing through the 2nd and 3rd *left* nipple regions. Owing to the position of the mice the interscapular lobe of the second nipple area was further distant from the applicator than the rest of the treated site. Since the interscapular lobes meet in the mid-dorsal line, the dose to the two lobes at this site was about equal and somewhere between a third and the full dose.

### *Examination of mammae.*

All nipple areas were examined with a binocular dissecting microscope unless the mice had died unexpectedly and decomposition was too advanced for study. Three such mice have been omitted from the final count of survivors beyond $11\frac{1}{2}$ months old, one for each of the doses used, although it can be stated that they did not have palpable tumours. Results have been based on examination with the dissecting microscope and it would have been impossible to exclude the presence of small growths in such decomposed and partly eaten animals. Right and left nipple areas were dissected out and fixed separately in Bouin's fixative. All were stained in bulk and examined as previously described (Pullinger, 1947,

1949). As a rule 1st, 2nd and 3rd nipple areas of both sides were mounted and kept. The 4th and 5th, which are bulky, were preserved only if they differed materially from the 1st. The 1st, 4th and 5th pairs of nipple areas served as indicators of circulating ovarian hormone. The irradiated mammary epithelium might have responded similarly to this hormone but in the event did not owing to vascular damage or to atrophy, or both. Microscopic sections were made of all but 2 tumours and a few were grafted.

### RESULTS.

Results relating to tumour incidence are summarised in Table I.

TABLE I.—*Mammary Tumours in Unmated RIIIb Mice after Local Surface and Penetrating X-irradiation of Second and Third Nipple Regions.*

| | | Number of mice. | | | |
|---|---|---|---|---|---|
| No. of experiment. | Total dose in r. | Treated. | Alive at $11\frac{1}{2}$ months. | With carinoma. | With benign growths. |
| II | 6000 | 14 | 6 | 1 | 0 |
| III | 4800 | 12 | 10 | 4 | 5 |
| IV and V | 3000 | 23 | 11 | 4* | 6 |
| Control | None | None | 21 | 0 | 1 |
| Control | None | None | 33 | 0 | 5 |

* Includes one which died at 8 months old.

Experiment I, which is not included here, was done to determine the dose level.

Experiment II: 6000 r in 10 weekly doses of 600 r was given to 14 mice aged 3 months. One mouse, No. 10, killed at 20 months old, had a mammary carcinoma. The 2nd right nipple area contained a small extraductal growth and also some intraduct growth near the nipple (Fig. 1). A small rectangular piece of tumour was cut out for section and proved to be an alveolar carcinoma. Two sarcomas. had developed in another mouse by $9\frac{1}{2}$ months of age.

Experiment III: 4800 r in 8 weekly doses of 600 r was given to 12 mice aged 4 to $4\frac{1}{2}$ months. Mice numbered 4, 10, 11 and 12 developed mammary tumours and number 10 a sarcoma as well.

Mouse 4 was killed aged $14\frac{1}{2}$ months. A slowly growing keratinising squamous-celled carcinoma was palpable in the 2nd right interscapular nipple area. Grafts from it survived in RIIIb males for 8 months. When examined only one had increased appreciably in size, though all had vascularised and were slightly larger.

Mouse 10 was killed aged $20\frac{1}{2}$ months. A large polymorphic-celled sarcoma was found in the right side of the thorax. In the interscapular lobe of the 2nd *left* nipple area there was a small palpable tumour which proved to be an alveolar carcinoma. A main duct of the same nipple area was filled with growth, also several smaller ducts at its anterior border. Sections of these showed early intraduct growth which had arisen *in situ* and penetrated the duct wall in places (Fig. 2).

Mouse 11 was killed aged $12\frac{1}{2}$ months. The 2nd right nipple area contained a rapidly growing alveolar carcinoma in the axilla, approximately $1 \times 1\frac{1}{5}$ cm. It was grafted and grew in 6 out of 6 RIIIb males.

Mouse 12 died aged 16½ months. A small carcinoma consisting of intra- and extra-duct growth was found by dissection in the 2nd right nipple area. Acinus formation could be seen at 100× magnification within one of the solid ducts of the whole mount (Fig. 3).

Experiment IV : 3000 r in 5 weekly doses of 600 r was given to 14 mice aged 4 to 4½ months old.

Mouse 22 died aged 8 months in an epidemic due to *B. piliformis* which killed all but 2 of this group. In the 2nd right nipple area in the isthmus between the proximal and distal interscapular lobes, there were 2 large nodules of acinar proliferation, one of which was dense and spherical resembling a small carcinoma rather than a benign tumour. The other of the two might have been either benign or malignant (Fig. 4).

Experiment V was done to supplement losses in IV. 3000 r in 5 weekly doses of 600 r was given to 9 mice aged 4½ to 5 months. Mice numbers 27, 30, and 33 developed mammary tumours.

Mouse 27 was killed aged 22 months. A small tumour was revealed by micro-dissection in the isthmus of the 2nd *left* nipple area (that is between the interscapular, axillary and proximal lobes). Sections of a dense portion of the tumour confirmed that it was an adenoacanthoma. This nipple area had received a dose of approximately 1000 r.

Mouse 30 was killed aged 20 months. There was a palpable mammary tumour in the mid-interscapular line. On dissection the greater part of it lay on the left of the mid-line having pushed aside the ducts of the 2nd left nipple area. The growth had arisen on the right side. It was an adenoacarcinoma about 0·6 cm. in diameter.

Mouse 33 was killed aged 11½ months. There was a palpable tumour in the 2nd right nipple region in the isthmus and distal interscapular lobe. It was an adenoacanthoma about 1 cm. in diameter.

Besides these malignant tumours there was an increase of apparently benign mammary growths in irradiated animals as compared with untreated controls (Table I). There were also many much smaller acinar and intraduct proliferations. The latter have only rarely been found in untreated mice whether unmated or breeders. The relationship of three of the actual tumours to ducts suggests the possibility of an origin from them. The majority of intraduct proliferations and benign growths, like the majority, 7 out of 9, of malignant tumours occurred on the right side exposed to maximum irradiation. For the doses 3000 r and 4800 r the maximum appeared to be the most effective for tumour induction. Losses in the experiment using 6000 r were too great to judge the effectiveness of this dose and are attributed to the younger age of the mice used and possibly too high a dose.

Three sarcomas occurred in 2 mice. There was no increase in lymphomas and no skin tumours arose. A few mice developed linear ulcers in the treated skin on the right side in later months of Experiments II and III. The epidermis was atrophic in that hair was scanty but the greater part of the surface epithelium was hyperplastic in section. Glücksmann (1951) together with Boag produced a few skin tumours by local treatment of skin in C57 black mice with a single exposure to an electron beam and dose of 7900 rep.

No ovarian tumours were found. The ovaries became atrophic but oestrus cycles were maintained in all those examined at a year old.

The mammae of 17 mice derived from all experiments were examined in early stages before the appearance of the first tumours. Atrophy was found in right second and third nipple areas by the fourth month after irradiation had been started. It was judged by reduction in lateral branching at first combined with shrinkage of the nipple area later. It was found in all of 11 mice examined at 8 months old. Involution, with which atrophy might be confused, does not begin to appear in unmated females of this strain until 10 to 11 months old. In addition 12 mice were killed for observation after 2 irradiations of 600 r each, 2 weeks after the first. By that time the mice were $4\frac{1}{2}$ to 5 months old. The treated right nipple areas were either unchanged or showed some degree of atrophy. At no time was there any hyperplasia beyond the degree of lobular alveolar development which is characteristic of at least half of the normal young unmated females of this strain.

During the course of irradiation vascular damage was done in the lungs but recovery took place. The treated nipple regions on the right side were more firmly adherent to subcutaneous tissue and to one another than normally and in consequence were less readily dissected without damage. A slight contraction of the right side of the thorax was noticeable. At one year old the weight of survivors in Experiment II was the same as in untreated controls.

### COMMENT.

It is clear that carcinoma arose from acinar and intraduct epithelium as a result of X-irradiation in the absence of ovarian tumours or of mammary hyperplasia. The new growths were accompanied by atrophy which began to appear earlier than 5 months old and was found in all mice by 8 months. Atrophy was probably due to some degree of vascular damage short of complete occlusion because the ducts persisted. In that case circulating oestrogen which was sufficient to keep up oestrus cycles and to maintain untreated nipple areas in a normal state could have reached the atrophic regions. Yet there was no response to be seen in them indicating that the atrophy was irreversible. Eight of the 9 carcinomas were found in mice older that 8 months so that it can be said that the conditions responsible for atrophy of neighbouring cells did not prevent subsequent growth, an observation which is in accord with human and experimental pathology. No conclusion can be drawn concerning the time or state of the mammae when the tumours were actually initiated. One of the tumours (on the left side) occurred after exposure to approximately 1000 r while atrophy had not begun in all mice after 1200 r. The necessity for a two stage experiment with two different agents is again apparent.

### SUMMARY.

1. Mammary carcinoma was induced in 9 out of 27 unmated RIIIb female mice without milk factor by local surface and penetrating X-irradiation of second and third nipple areas. The growths arose from intraduct and acinar epithelium. No spontaneous carcinoma occurred in unmated females of this strain. Less than 2 per cent were found in breeders.

2. Growth of tumours was preceded by atrophy but the sequence of events concerning the time of onset or initiation of actual neoplastic change could not be ascertained. The atrophic process did not prevent subsequent growth of the tumours. Hyperplasia was not seen at any stage.

3. No ovarian or skin tumours arose, nor was there any increase in lung adenoma or in lymphoma. There were 3 sarcomas.

The standardised nomenclature of the mouse strain used is RIIIfB/Pu.

I am greatly indebted to Dr. P. R. Peacock, the Board of Management of the Royal Beatson Memorial Hospital, and to Dr. J. M. Glennie for generous allocation of space and facilities to do these experiments.

It is a pleasure to acknowledge the enthusiastic assistance of Dr. A. F. Howatson concerning technique and dosage and to thank Mr. J. B. McGregor for his supervision of the X-ray unit during treatments. The illustrations are due to the skill and persistence of Mr. P. R. Price with this difficult photographic subject.

Valuable preliminary experience of dosage was gained through the kind co-operation of Mr. A. K. Powell, Mr. D. E. A. Jones and Mr. P. Tothill of the Mount Vernon Hospital, Northwood.

### REFERENCES.

Friedewald, W. F., and Rous, P.—(1944) *J. exp. Med.*, **80**, 101.

Glücksmann, A.—(1951) *J. Path. Bact.*, **63**, 176.

Lorenz, E., Eschenbrenner, A. B., Heston, W. E., and Uphoff, D.—(1951) *J. nat. Cancer Inst.*, **11**, 947.

Pullinger, B. D.—(1947) *Brit. J. Cancer*, **1**, 177.—(1949) *Ibid.*, **3**, 494.

### EXPLANATION OF PLATES.

Fig. 1.—Experiment II, Mouse 10. Right (*a*) and left (*b*) second nipple areas. The right contained a small extra- and intraduct carcinoma marked 1. Atrophy and shrinkage are conspicuous in the right. Approximate position of left nipple marked n. × 3.

Fig. 2.—Experiment III. Mouse 10. (*a*) Right second and third nipple areas have been pushed apart by a sarcoma found in position s. The second right nipple was near n. (*b*) The second left nipple region contained a carcinoma 1 in the interscapular lobe, intraduct growth in a main duct 2 and in ducts cut out from 3. Atrophy is conspicuous on both sides except around the left nipple n. × 3.

Fig. 3.—Experiment III, Mouse 12. Right second nipple area. A small extra- and intraduct carcinoma is shown at 1. Intraduct acinus formation could be seen at 100 × magnification. × 3.

Fig. 4.—Experiment IV, Mouse 22. Right second nipple area. A small adeno-carcinoma is shown at 1. Atrophy had begun by 8 months. Lateral branching and lobular-alveolar differentiation of the other nipple areas were much more developed. × 3.

The unmarked black areas are masses of fat in which the gland is embedded.

Fig. 1, left nipple area is indistinguishable from a normal involuting second nipple area of the same age.

Pullinger.